# The File

**Published:** 1859-11

**Authors:** J. G. Hamill

**Affiliations:** Lancaster


### " THE FILE."

No instrument employed by our profession has been so effectually cursed by those, both in and out of the profession, ignorant of its proper uses, as the file. In fact, it was for a long time considered, by a large portion of almost every community, the very "Shibboleth" of destruction to the teeth. But notwithstanding the odium cast upon it, men, with honesty of purpose as their motto, continued to use it; and now its utility is acknowledged by most communities, by permitting its use upon the teeth whenever their dentist thinks it is required. But this is not universally the case. There are still some benighted spots, that have never enjoyed the blessing of an honest man, *educated* in the dental profession. Such localities are generally prevented from having such a man, by half-a-dozen or more *excuses* for men, who travel over the country and solicit work—doing it so cheap—it is "dogged cheap," to every body except the man that pays for it.

In such a community, if the judicious operator attempts to do his duty, by making use of the file to any extent, the patient shrinks back with "holy horror" at the very thought of it: and the oft-repeated absurdity "of a whole denture having been lost because one tooth had been filed" is hurled at him with defiance. Truly such people need our sympathy, yet still it is not much better than "throwing pearls before swine" to extend it where *blissful ignorance* reigns supreme. But these things are "all in a man's life time," and are rather to be looked for in "the course of events." The exertions of a good operator, together with the touchstone of time, will dissipate them forever.

In this world in general, as any thing is good is it liable to abuse. The file and cutting instruments are not an exception to this rule by any means. We must, therefore, use them with clear judgment, and with honest convictions that we are putting the teeth we are operating upon in a better condition to resist decay, than could be done by any other treatment. If a tooth needs filling, it will not do for us to catch up a file and cut away, with no other object in view than simply gaining room to fill it; but we must examine all the surroundings before we begin. We must note the relative position the teeth sustain to each other, as they stand, before we begin to operate, and ascertain, if possible, how much influence such position had in the production and progress of the decay. We must form an opinion as to what the benefits accruing to these teeth will be, provided they shall be cut in this, that, or the other manner. We must also consider the habits of the individual we are to operate for, whether he or she will be likely to pay any attention to cleanliness of the teeth after they shall have been filed, or not. But above all things must we consider the physical characteristics of the teeth themselves. Every dentist should have the different classifications of the teeth fixed in his memory, so as to be able to decide at once as to the particular class to which any presented may belong, and thereby be able the more readily to decide upon their ability to resist decay. We will illustrate the uses we make of the file and cutting instrument (we consider these instruments together, because we almost always use them both upon the same tooth), upon two classes of teeth.

First, we will take that class which is considered the best that human flesh is heir to, viz: those in which the phosphate of lime largely predominates—whose crowns are short and thick, and the diameter of whose necks is almost as long as their cutting edges. When such teeth are presented for the removal of superficial decay, we do not hesitate to use the file and cutting instrument until we have removed it all. We do not leave a shoulder at the neck (although it might be done with more safety in this class of teeth than any other), as some say they do, for the reason that we do not consider that decay which is *superficial* requires enough filing for its removal to form a shoulder. We recognize that decay only as superficial which is confined to the enamel. The very moment, in our opinion, that the dentine becomes softened, then the decay ceases to be superficial. When the decay has progressed too far to be recognized as superficial, we prefer doing as Dr. Forbes, of St. Louis, says he does—throw the responsibility upon the patient, and not lull him into apparent security by filing the tooth. In removing the decay, we file the surface of the tooth rounding, leaving it as near the shape it had before decay began as possible. It is reasonable, that a round surface will be less likely to retain foreign substances than a flat one; especially will it be so if there are two such surfaces placed together. But the whole procedure thus far will be suicidal to the teeth filed, unless the surface be left perfectly smooth and polished. If this class of teeth require filling, we file away until we get room enough to manipulate with ease. In shaping the cavity, we are careful to have points for retaining the filling in those parts of it next the neck of the tooth and the cutting edge.

After consolidating the filling properly, we file off the edges of the tooth and the gold all together, so as to leave a rounded surface, as above described in the case of superficial decay. We leave a shoulder at the neck of this class of teeth, when we prepare them for filling. The points for retaining the filling, noticed above, are worthy of note; because, if they were located at any other part of the cavity, there would be danger of injuring them with the file while giving the tooth a rounded finish, and thereby endanger the integrity of the filling.

The second class of teeth which we wish to notice must be approached altogether differently. We refer to that class which is characterized by long, slender crowns, white color, and less density of structure than the one we have already noticed. They are also much broader proportionally at the cutting edges than at the margin of the gum. They rest against each other only near the cutting edges, forming spaces below this point of contact of an inverted V shape.

When such teeth are to be operated upon for the removal of superficial decay, and that decay is below the point at which the teeth rest together, we use a sharp-pointed file, with one side flat and the other convex—the convex side being the cutting one. The natural spaces between these teeth will generally allow the motion of such a file without destroying the enamel where they rest against each other; but if the space is not large enough to admit the file, we obtain such space by wedging, then file the decay away, polish the surface, and allow the teeth to come together again. But if the decay has attacked these teeth at the point where they rest together, we file extensively, cutting away even more than is really necessary for the removal of the decay. In this operation, we direct the file so as to cut mostly from the lingual side of the teeth. We only remove enough from the labial side to obtain an edge strong enough to bear the manipulation of the burnisher in putting a polish upon it. We follow about the same plan when these teeth need filling, but carry it as much farther as is required for performing the operation properly.

This class of teeth, as is well known, always come together after they have been operated upon, let the operator file as he pleases, unless all in the front part of the mouth be filed at the same time. Therefore, both in the removal of superficial decay and in the preparation for filling, we bevel off the lingual edge of the filed surface, making it rounding; but the labial edge we leave almost square, so that when the teeth come together again, only about a line and a half of the labial edge of each tooth will be in contact with the other, and that near the cutting edge. When the teeth have assumed their places again after having been filed in this way, the spaces inside between the beveled edges, and also between the teeth themselves below the point of contact, should be large enough to allow the tongue, brush or toothpick to remove everything that would be likely to induce decay again.

Of course it will be understood that what we have said refers only to the approximal surfaces of the incisor and canine teeth.

At the last meeting of the American Dental Convention, the discussions upon this subject took a broad and general direction, irrespective of the different classes of teeth which are met with. We hope that the Executive Committee, when laying out work for the next meeting, will classify the teeth, and call upon the members for their mode of operating with the file and cutting instrument upon each class respectively.

LANCASTER, OCT. 1, 1859. J. G. HAMILL.

